# SEM/EDX analysis of stomach contents of a sea slug snacking on a polluted seafloor reveal microplastics as a component of its diet

**DOI:** 10.1038/s41598-022-14299-3

**Published:** 2022-06-17

**Authors:** Giulia Furfaro, Marcella D’Elia, Stefania Mariano, Egidio Trainito, Michele Solca, Stefano Piraino, Genuario Belmonte

**Affiliations:** 1 Department of Biological and Environmental Sciences and Technologies - DiSTeBA, University of Salento, Via Prov.le Lecce‐Monteroni, 73100 Lecce, Italy; 2 Department of Mathematics and Physics “Ennio de Giorgi”, University of Salento, Via Prov.Le Lecce-Monteroni, 73100 Lecce, Italy; 3 Marine Protected Area ‘Tavolara-Punta Coda Cavallo’, Olbia, Italy; 4 Museo di Biologia Marina “Pietro Parenzan”, Via Vespucci 13/17, Porto Cesareo, 73010 Lecce, Italy; 5 Consorzio Nazionale Interuniversitario per le Scienze del Mare (CoNISMa), P.le Flaminio 9, 00198 Rome, Italy

**Keywords:** Biological techniques, Biophysics, Biotechnology, Ecology, Zoology, Climate sciences, Environmental sciences

## Abstract

Understanding the impacts of microplastics on living organisms in aquatic habitats is one of the hottest research topics worldwide. Despite increased attention, investigating microplastics in underwater environments remains a problematic task, due to the ubiquitous occurrence of microplastic, its multiple modes of interactions with the biota, and to the diversity of the synthetic organic polymers composing microplastics in the field. Several studies on microplastics focused on marine invertebrates, but to date, the benthic sea slugs (Mollusca, Gastropoda, Heterobranchia) were not yet investigated. Sea slugs are known to live on the organisms on which they feed on or to snack while gliding over the sea floor, but also as users of exogenous molecules or materials not only for nutrition. Therefore, they may represent a potential biological model to explore new modes of transformation and/or management of plastic, so far considered to be a non-biodegradable polymer. In this study we analysed the stomachal content of *Bursatella leachii,* an aplysiid heterobranch living in the Mar Piccolo, a highly polluted coastal basin near Taranto, in the northern part of the Ionian Sea. Microplastics were found in the stomachs of all the six sampled specimens, and SEM/EDX analyses were carried out to characterize the plastic debris. The SEM images and EDX spectra gathered here should be regarded as a baseline reference database for future investigations on marine Heterobranchia and their interactions with microplastics.

## Introduction

Despite increased attention the unceasing accumulation of plastic materials in aquatic environments represents a critical issue and emergent threat worldwide—boosted by an increasing global plastic production and the improper disposal of plastic waste. The impacts of plastic on marine organisms are widely reported^1–3^ and the list of threatened organisms is constantly increasing, as witnessed by the high number of recent scientific papers on this topic^4–6^. Plastic items can be categorized according to their size as: mega- (> 1 m diameter), macro- (between 2.5 cm and < 1 m), meso- (between 5 mm and < 2.5 cm), micro- (between 0.1 μm and < 5 mm) and nanoplastics (< 0.1 μm)^7–13^. Focusing on microplastic, a great effort is currently being made in collecting data on its distribution, quantification, and the occurrence in biological taxa, in order to assess how advanced is the level of contamination of food webs in natural environments. In this framework, several protocols have been developed to optimize the extraction of microplastics from different marine environments and substrates (surface waters, water column, and seabed at different depths) or from different animal taxa, organs, and tissues (e.g., stomach, liver, kidney, gills, lungs, gonads, and specific tissues)^14,15^. To date, vertebrates have been the primary focus of investigation for the detection of plastic ingestion^16–21^. However, smaller plastic items are commonly ingested by a wider range of (smaller) organisms^7,22–24^. Major groups of invertebrates, such as annelids (Polychaeta), crustaceans (Cirripedia, Amphipoda) and echinoderms (Holothuroidea), are known to ingest microplastic particles during laboratory trials^25,26^. The common destiny of this ingested plastic seems to be expulsion within faecal material^25,27–30^.

The characterization of collected microplastics can take place using different techniques, but most used is Scanning Electron Microscopy (SEM) for morphological investigation, associated with Energy Dispersive X-ray spectroscopy (EDX) for elemental microanalysis. The combination of morphological and elemental composition analyses has proved an effective tool to identify plastic materials extracted from the stomach contents and the tissues of fishes, marine invertebrates, and sediments^31–34^. These two coupled techniques seem to be highly effective when ‘in situ studies’ (i.e., based on organisms freshly collected in the field, not subject to laboratory experiments) are carried out, and when other contaminants are present. Indeed, synthetic molecules and materials of anthropogenic origin can interact with the microplastics under investigation, leading to detection of emission spectra not comparable with those available in reference libraries. This is the main drawback of other techniques, such as the Fourier transform infrared spectroscopy analysis (FTIR), requiring comparative screening of reference spectra to identify the chemical nature of the sample. This limitation does not apply to SEM/EDX, which is also a faster technique with the potential to analyse many samples in a relatively short time^33,35–37^. Conversely, procedures of FTIR spectroscopy are longer, leading to analysis of just a sub-sample of the total extracted microplastics^31,38,39^, with a consequent decrease of accuracy (e.g., due to wrong identifications and/or underestimation).

The Mediterranean Sea is known as a hotspot of biodiversity, with high rate of cryptic diversity and new species being continuously revealed and described^40–44^. Unfortunately, it is also particularly sensitive to microplastic pollution, with contamination levels almost four times greater than the North Pacific Ocean, comparable to the five top accumulation zones known for subtropical ocean gyres^45,46^. A significant land-based plastic input in the semi-closed Mediterranean Sea is also combined with a proportion of floating plastics originated outside the basin^45^ and transported by a constant inflow of superficial waters from the Atlantic Ocean^47,48^. The patchy spatial distribution of floating plastics in the Mediterranean Sea suggests that shelf areas near river estuaries and population centres may be related to local plastic accumulation in short term, but the overall Mediterranean distribution of plastics seems largely dependent on the pattern of the surface circulation^45,48^. Eventually, nearly 94% of fragmented plastics accumulate on the seabed with a large proportion occurring as microplastics, up to 2175 items per Kg of sediment in areas subject to high anthropogenic pressures, such as the Venice Lagoon^49^.

The Mar Piccolo of Taranto (Apulian, Ionian Sea, Central Mediterranean Sea) is a semi closed basin divided in two sheltered and interconnected inlets, a natural site subjected to heavy human pressure and environmental pollution, as the consequence of the high number of industries and intensive farming of mussels and fish operating in the area. The Mar Piccolo contains more than 30 natural submarine springs of brackish water, and it receives some surface creeks from the surrounding territory^50^. This water input somewhat opposes the entrance of marine water, which is also limited due to a very weak tide oscillation (maximum 30 cm of daily excursion). Such negligible introduction of open sea water minimizes the load of any foreign litter and/or plastics^51–53^, so restricting plastic pollution to local and easily identifiable input sources^54^. Due to its geomorphological peculiarities, Mar Piccolo can be used as a model system, a natural laboratory where to observe and predict the negative effects that may occur on a larger geographical scale^53–55^, and even to propose localized recovery/remediation interventions.

Various papers have been recently published on the presence and the identification of microplastics from molluscs, but these were focused mainly on filter feeding animals such as bivalves and/or on a few detritivore organisms such as some gastropod species^32,56–59^. Unexpectedly, no studies focused so far on the occurrence of microplastics in marine Heterobranchia (Mollusca, Gastropoda), better known as sea slugs, usually living on the organisms on which they feed or gliding around on the sea floor in search for food. As far as we know, the only published paper on microplastics and sea slugs deals with laboratory experiments which did not involve analytical methods of microplastics analysis^24^. Marine Heterobranchia are generally small and characterized by a reduced or completely lost shell, a very variable body plan and ecological patterns, and a highly specialized diet^60–63^. They are known to have a unique tendency to use non-food ingested particles for other purposes, mainly defensive chemical strategies^64–70^. In fact, this group of molluscs shows a wide range of sophisticated defensive stratagems that evolved in response to the reduction or completely loose of the shell. They are able to acquire, accumulate and/or modify chemical compounds and/or entire intracellular organelles, extracting from their stomach contents and retaining them in a non-feeding role (e.g., for defensive purposes)^71–74^. This raises the question of whether these delicate slugs may have the potential to detect, handle and possibly modify traditional plastic polymers (e.g., non-biodegradable ones). Indeed, Heterobranchia are able not only to recover and accumulate chemical compounds (with different properties) from their diet but can also manipulate these chemicals by modifying them into new ones^75,76^. This capability also proved to be interesting for potential applications in pharmacology^77^.

Among heterobranchs, the family Aplysiidae (order Aplysiida) is characterized by relatively large species with a reduced or completely absent shell in adults. They have a more or less selective diet consisting mainly of plant organisms and accidentally of small animals living in the detritus^78^. Even if the interactions between microplastics and Aplysiidae are almost unknown, several studies have been done on other biological aspects like the ecological, behavioural, chemical, developmental biology and molecular, focused on this interesting heterobranch group^79,80^.

A resident population of the Aplysiidae species *Bursatella leachii* Blainville, 1817 lives in the Mar Piccolo of Taranto (Ionian Sea, Apulia, Italy) and it is characterized by seasonal demographic expansions perhaps due to an increase in the trophic availability, a trait shared with other heterobranchs species living in analogous habitats^81^. This species contains several chemical compounds with different biological activity^82–85^, may reach up to 10 cm in length and shows flexibility in prey selection even if it is far from being considered as a ‘non-selective’ bottom feeder^86^. Given the large availability of plastic litter in the Mar Piccolo sediments, we aimed to: (1) investigate the potential occurrence of microplastics in the stomach contents of *B. leachii*, (2) collect data on the possible interaction with microplastics, (3) characterize the detected microplastics using SEM/EDX analyses for the first time on Heterobranchia, and (4) create a first preliminary repository of SEM/EDX’s images and microplastic spectra obtained from environmental samples that is ‘non-virgin microplastics’. These data will help to deliver a reference baseline of microplastic contamination for future monitoring studies, not only in areas under severe contamination levels as in the Mar Piccolo of Taranto, but also in less polluted areas under chronical, long-time exposure.

## Materials and methods

**Figure 1:**
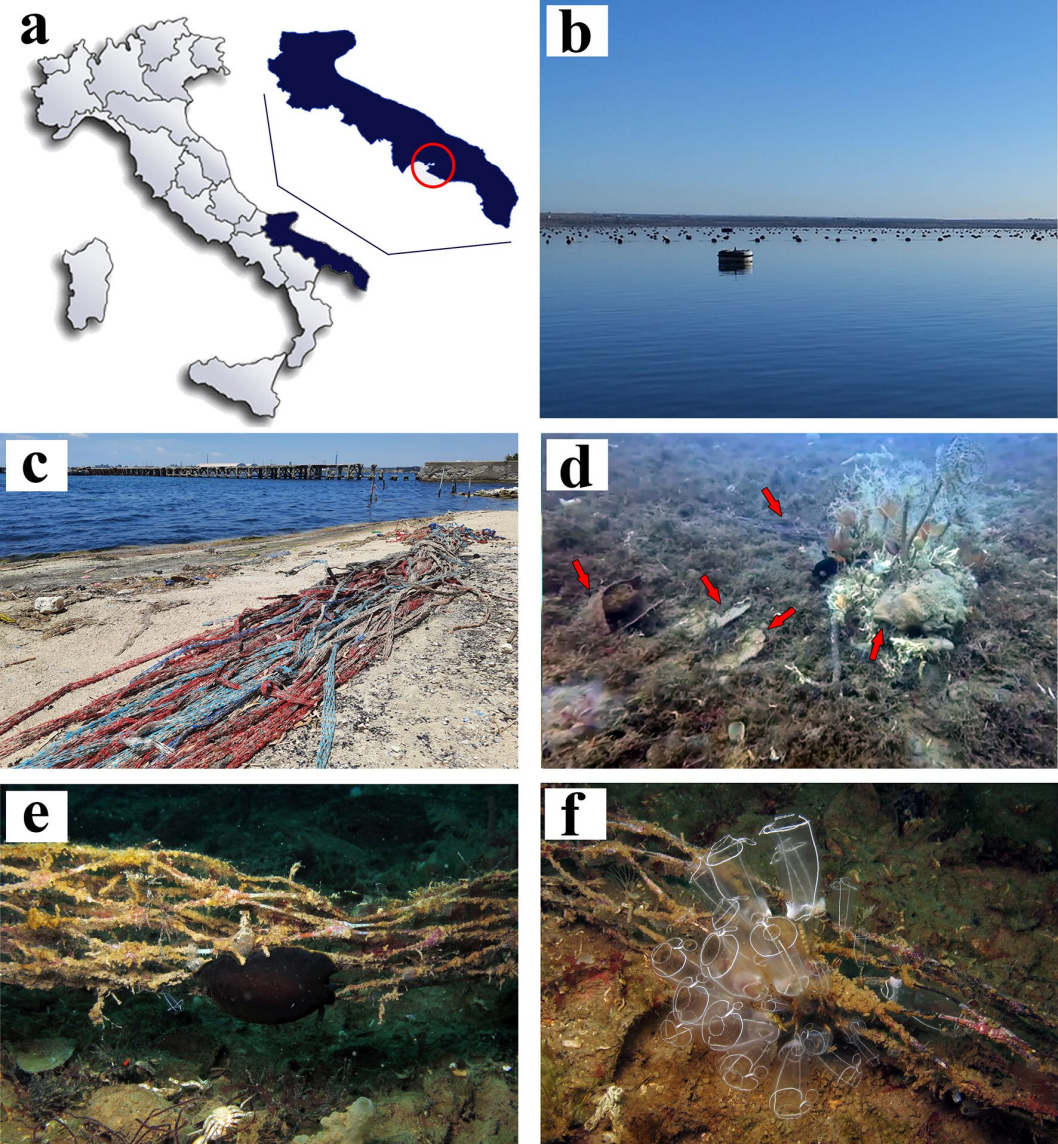
Sampling locality objects of the present study. (**a**) Map of the sampled area in Apulian Ionian Sea with the Mar Piccolo highlighted by the red circle (Taranto, Southern Italy). Map was obtained using Microsoft Paint 3D Version 6.2105.4017.0. (**b**) External photo of the buoy indicating the submerged underwater site (40° 29′ 05.1′′ N 17° 15′ 08.3′′ E). (**c**) Fishing nets along the beach. (**d**) Polluted environment characteristic of Mar Piccolo (9 m depth). (**e**) Fishing net with the sea slug *Dendrodoris limbata* (Cuvier, 1804) crawling on it. (**f**) Sessile tunicate, *Clavelina lepadiformis* (Müller, 1776), living on a fishing net in the sampling site. Red arrows indicate the plastics present in the studied area.

The sampling locality was in the Mar Piccolo (40° 48′ N, 17° 25′ E) in Taranto (Apulian, Ionian Sea, Central Mediterranean Sea), a semi closed highly polluted basin, hosting a high number of industries and intensive farming of mussels and fish (Fig. 1).

**Figure 2:**
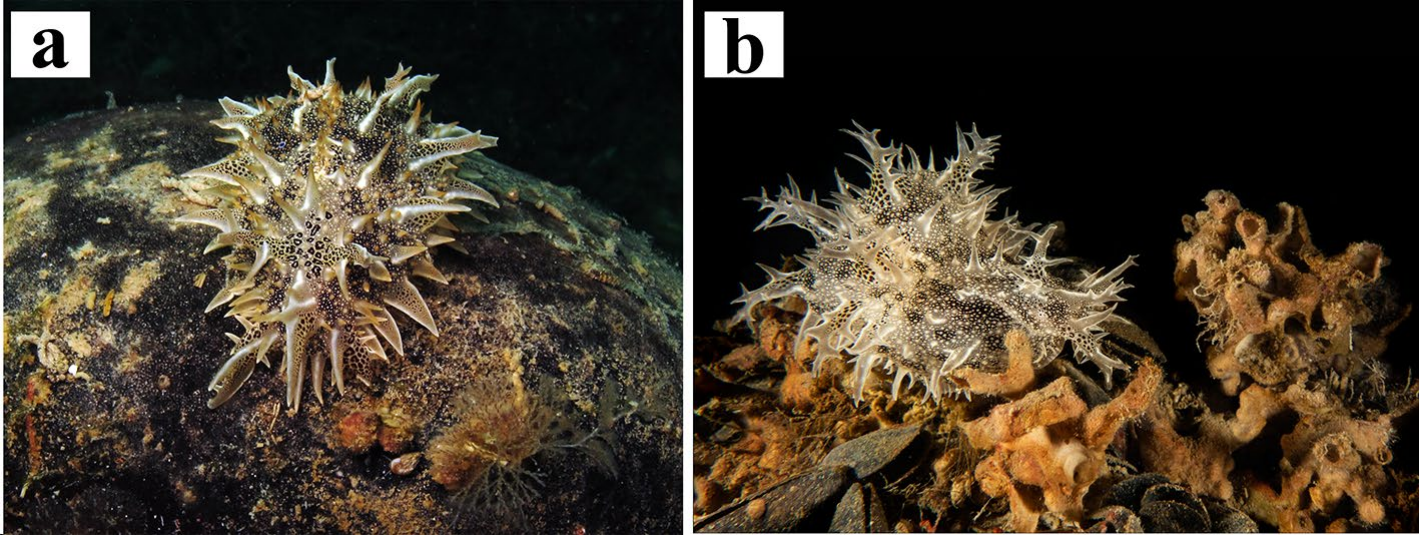
Species objects of the present study. (**a**,**b**) In situ images of *Bursatella leachii* specimens.

Six specimens of *Bursatella leachii* (Fig. 2) were collected by scuba diving at 10 m depth (Fig. 1a,b) and identified according to the external morphological diagnostic characters^87,88^.

Each sample was observed in situ and in laboratory, photographed using a stereomicroscope and a microscope, preserved in 95% ethanol for future analysis and deposited as voucher at the Department of Science of the Roma Tre University (Rome, Italy). To reduce possible contamination of samples, with the consequent overestimation of microplastic detected, preventive measures were applied. In particular, specimens were manipulated underwater without using gloves, wrapped in aluminium foil before having been placed in a tank and finally transferred to the laboratory where they were suddenly stored in alcohol and analysed. Furthermore, in each step of the laboratory analyses, only glass materials washed with micro-filtered water were used.

### Anatomical dissection

Analyses of the internal anatomy of the collected specimens were carried out by anatomical dissection under the stereo microscope at different magnification levels. The digestive system, from the oesophagus (taken right at the end of the gizzard) to the terminal anus, was isolated from the rest of the body and prepared for the next microplastic extraction protocol. Stomachal content was observed at the stereomicroscope and the ingested particles that were undoubtedly not plastics were separated for further detailed observations. The rest of the stomachal content, including visible fibres and microplastic debris, was placed in a separate 50 ml tube.

### Microplastic extraction and samples preparation

Prior to carrying out the microplastics extraction and characterization, the digestive system of each specimen was rinsed with pre-filtered (0.22 μm) deionized water and centrifuged to eliminate alcohol used to store them. Subsequently each pellet was incubated with 10% of KOH (w/v) solution prepared using KOH pellets (Sigma-Aldrich, Saint-Quentin-Fallavier, France) and double-distilled water. Then they were placed on an agitation plate (IKA RT15, Staufen, Germany) set at 300 rpm and 60 ± 1 °C for 24 h. After digestion, all samples were filtered on 90 mm diameter GF/C glass microfibre filters (Whatman, Velizy-Villacoublay, France) using a vacuum system. Filters were then placed in closed Petri dishes until subsequent analysis. For the first characterisation, filters were observed under a stereomicroscope (Nikon SMZ25, Tokyo, Japan), allowing the identification of plastic particles. Items with characteristics similar to plastic polymers were characterized by size (< 100 µm; 100–500 µm; ˃500 µm) and colour. Filtered fragments and fibres were then rinsed in distilled water and mounted on double-sided adhesive carbon tabs on aluminium SEM stubs for successive SEM/EDX analyses.

### SEM/EDX analyses

SEM/EDX analysis was conducted on individual candidate microplastics selected by optical microscopy from the glass microfibre filters through which the treated *B*. *leachii* guts were filtered. SEM/EDX allowed many potential microplastic particles to be screened in a relatively short time. SEM/EDX screening utilized surface morphology and elemental composition to determine whether each particle was potentially a plastic. The analyses were conducted using two different microscopes: The JSM-6480LV Scanning Electron Microscope (JEOL Ltd., Tokyo, Japan) with a Sirius SD Energy Dispersive X-ray Spectometer (iXRF Systems Inc., Houston, USA) (hereafter as SEM-JEOL) and the Sigma 300 VP Field Emission Scanning Electron Microscope (ZEISS, Oberkochen, Germany) (hereafter as FESEM-ZEISS). The former was used to the preliminary morphological assessment and to carry out the detailed microanalysis while the latter to obtain high resolution images useful to the in-depth observations of the morphological details. After checking that there was no sample charging under the electron beam, to avoid the contamination due to chemical artifacts introduced by the metal coating of the samples, these latter were initially analysed without the gold-coating step. The SEM-JEOL provided low resolution imaging of particle surface structures (not shown), as well as elemental composition signatures. Spectra of the chemical composition of the debris analysed were then compared with those already present in literature and related not only to microplastics but also to other organic compounds that cannot be removed by treatment with the KOH solution (like for example cellulose and chitin) but that could anyway be ingested by *B. leachii*.

To obtain high resolution SEM images, fragments and fibres previously mounted on the aluminum stubs and analysed with EDX, were afterwards gold coated in an Emitech K550x sputter unit, and finally examined by FESEM-ZEISS up to × 5000 magnification. The integration between results obtained by SEM and EDX methods and taking into consideration that samples were previously digested with 10% of KOH (w/v) solution which means that a lot of organic compounds were consequently excluded from the dataset under investigation, allows establishing if the analysed samples were effectively plastics or not.

### Compliance with ethical standards

All applicable international, national and/or institutional guidelines for sampling, care and experimental use of organisms for the study have been followed and all necessary approvals have been obtained.

## Results

**Figure 3:**
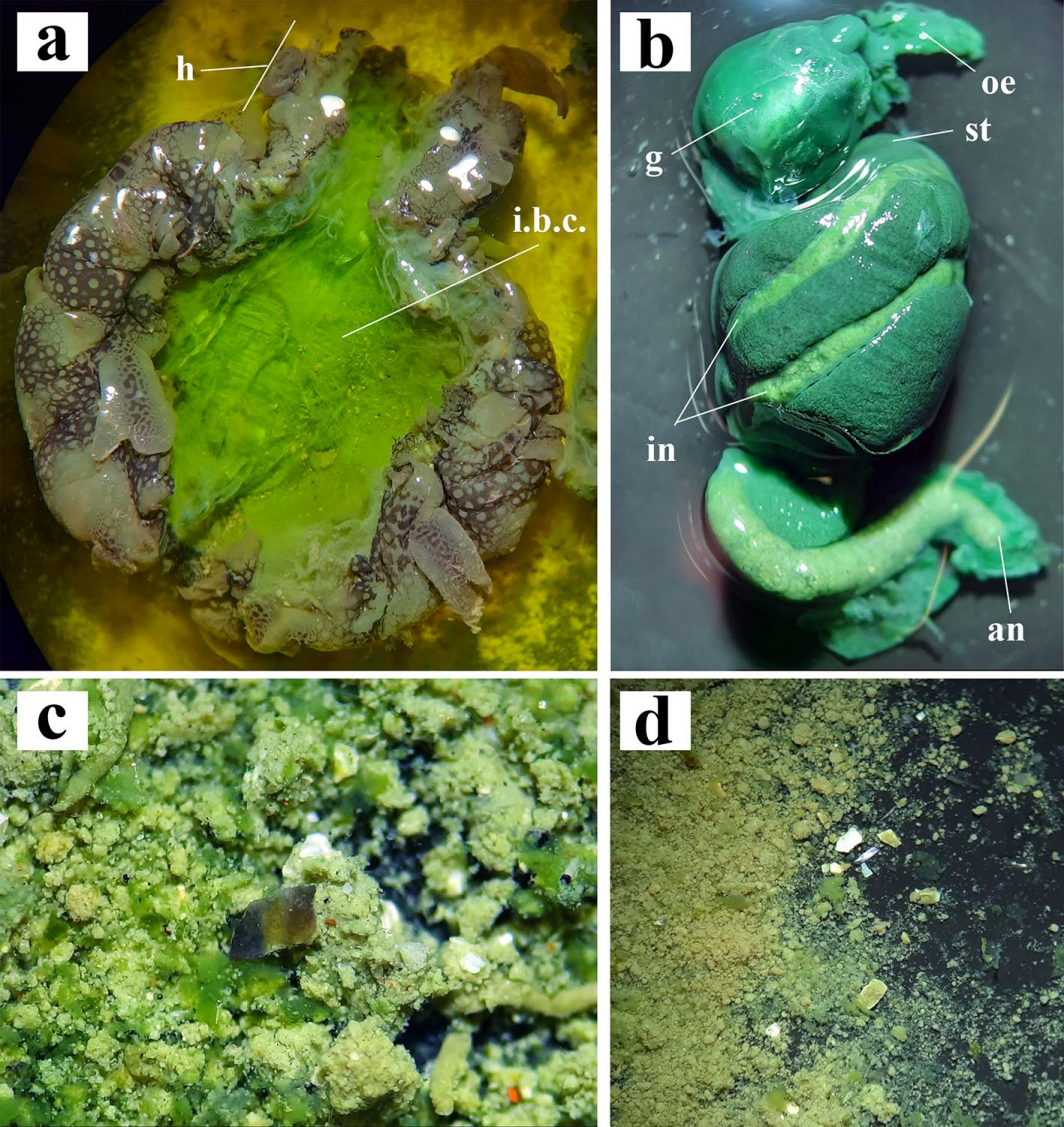
Anatomical dissection of *Bursatella leachii*. (**a**) Dorsal view of the body after removal of the internal visceral mass. (**b**) The digestive apparatus (oesophagus, gizzard, stomach, intestine, anus) separated from the rest of the body. (**c**,**d**) Images showing the stomach content where are visible the small microplastic debris. *an* anus, *g* gizzard, *h* head, *i.b.c.* internal body cavity, *in* intestine, *oe* oesophagus.

**Figure 4:**
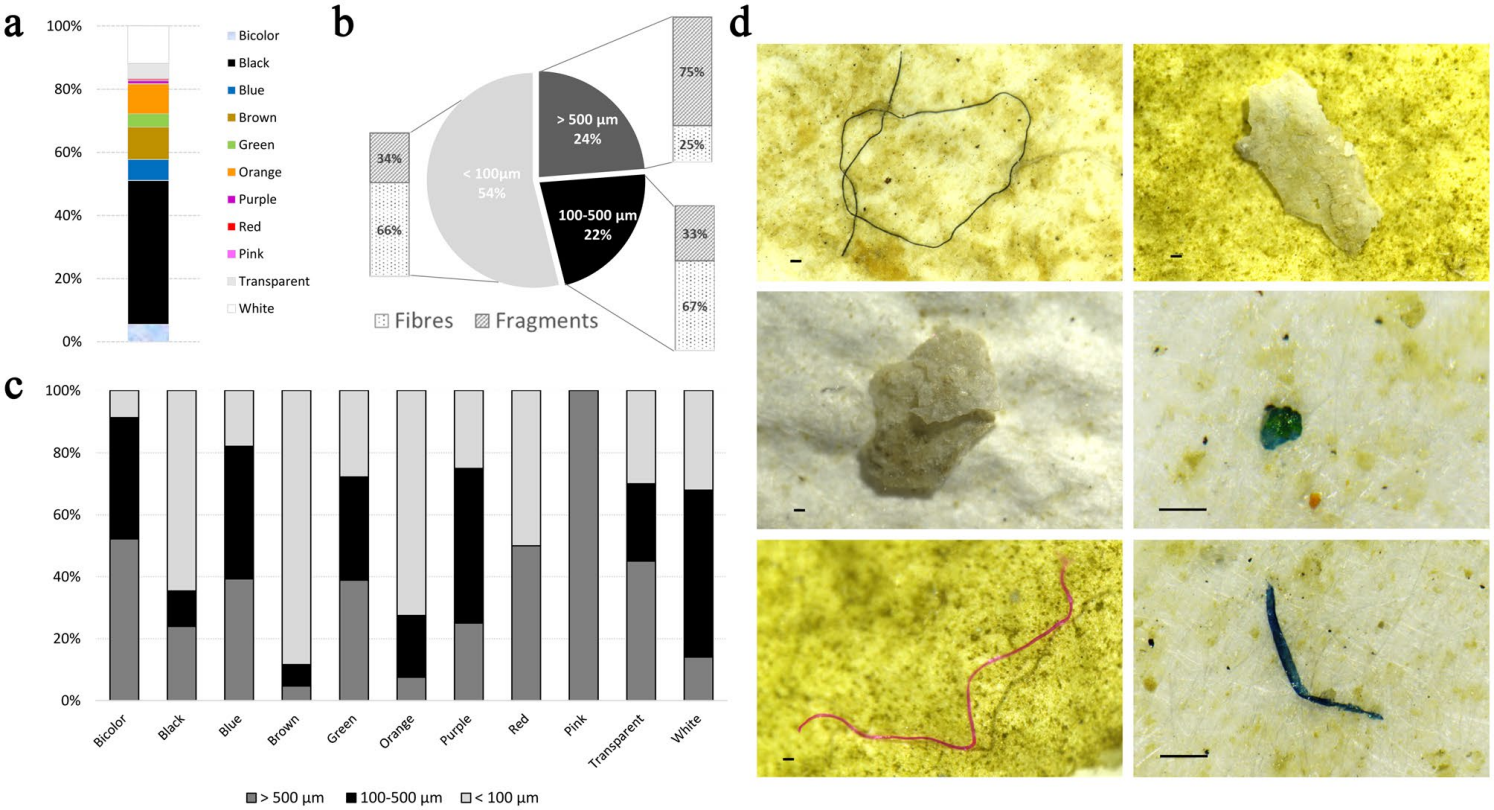
Relative abundance (%) of microplastics detected on glass microfibre filters after digestion and filtration of gastrointestinal tracts of six Nudibranchia and classified according to (**a**) colour; (**b**) size and shape and (**c**) size for each colour; (**d**) images observed by stereomicroscope of different shapes of MPs found in specimens. Scale bar = 100 μm.

Digestive systems from six individuals of *B. leachii* (Fig. 2a,b) from the Mar Piccolo of Taranto (Fig. 1b,c) were dissected and isolated (Fig. 3) for preliminary microscopic analyses. Microplastics were found in the stomach contents of all specimens (Fig. 4) and sorted according to their morphologies into fragments and fibres and by size and colour. Fibre particles were fewer than the number of irregular fragments in five out of six analysed stomach contents (Table 1).

**Table 1:** Voucher numbers of the specimens of *Bursatella leachii* from Mar piccolo (Taranto, Apulia) analysed with the relative number of fibres and fragments of microplastics detected.

Species	Voucher	Fibres	Fragments
*Bursatella leachii*	RM3_1890	21	36
	RM3_1892	13	27
	RM3_1893	16	85
	RM3_1894	61	52
	RM3_1895	16	18
	RM3_1896	20	56

### Microplastic extraction and analyses

In the gastrointestinal tract of six *B. leachii* individuals, many microplastics were detected, visible onto the glass microfilters (Fig. 4a-d). The number and type of microplastics detected in each analysed sample were reported in Table 1. The microplastics size distribution is presented in Fig. 4b. The size class distribution revealed a marked prevalence for particles smaller than 100 µm (54%), followed by particles with a range 100–500 µm (24%) and particles over 500 µm (22%). A total of 11 chromatic components were observed. The black colour dominated with 45.60% of particles found in specimens (Fig. 4a). Other colours representing important proportions were transparent (4.75%), white (11.87%), brown (10.67%), orange (9.92%), blue (6.65%), bi-colour (5.46%), green (4.27%) and small percentage of the other colours. In addition, we reported the size distribution for each colour, as shown in Fig. 4c. The particle sizes varied for every colour, without a predominance of size in the case of the most common colours. Only the small percentage of pink fibres (0.23%) observed on the filter had a length over 500 µm, while the brown particles reached mostly 500 µm.

### SEM/EDX analyses

**Figure 5:**
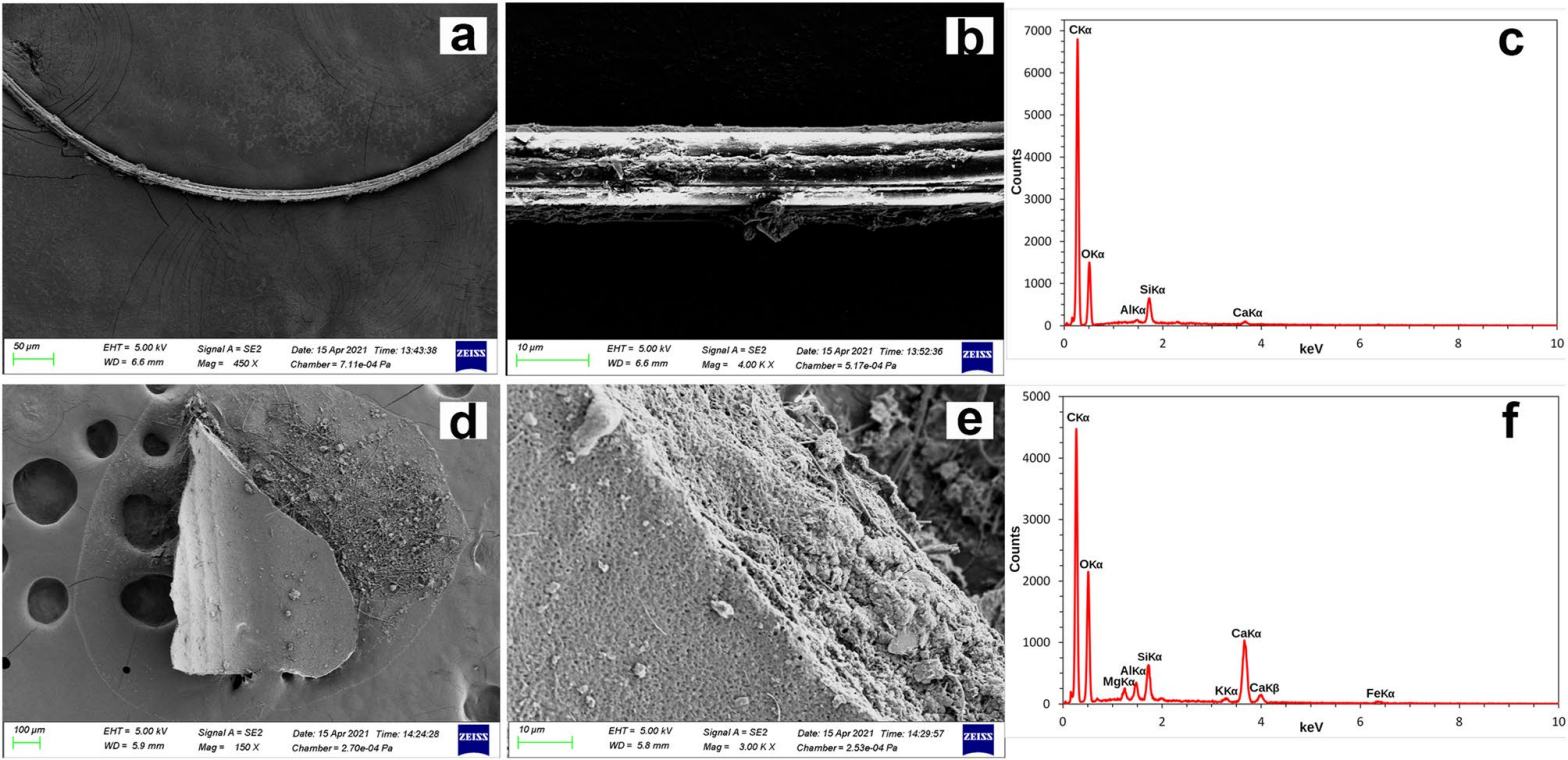
SEM/EDX images and microanalysis from canonical microplastic. SEM images (**a**,**b**,**d**,**f**) and EDX spectra (**c**,**f**) from a typical microplastic fibres (**a**–**c**) and typical microplastic debris (**d**–**f**) extracted from the stomachal content of the collected *Bursatella leachii* specimens.

**Figure 6:**
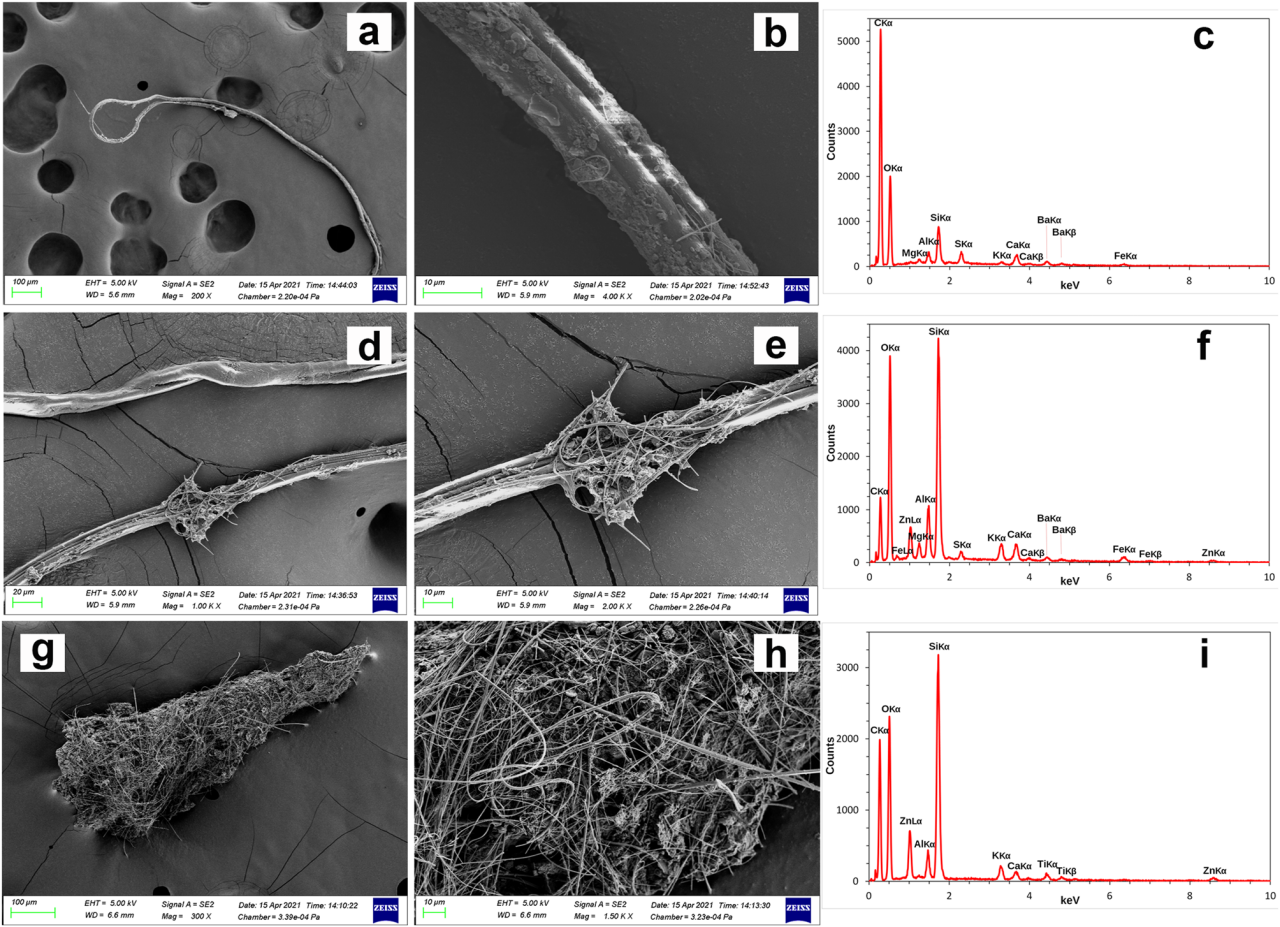
SEM/EDX images and microanalysis from different kind of plastics. SEM images (**a**,**b**,**d**,**e**,**g**,**h**) and spectra (**c**,**f**,**i**) showing the different kind of microplastics obtained from *Bursatella leachii* analysed. (**a**–**c**) Fibreglass fibre containing Carbon and Oxygen as the most abundant elements. (**d**–**f**) fibReglass fibre showing the Silicon peak and Carbon, Oxygen and several other minor elements. (**g**–**i**) Fibreglass debris with the Silicon peak followed by Carbon and Oxygen and several other minor elements.

**Figure 7:**
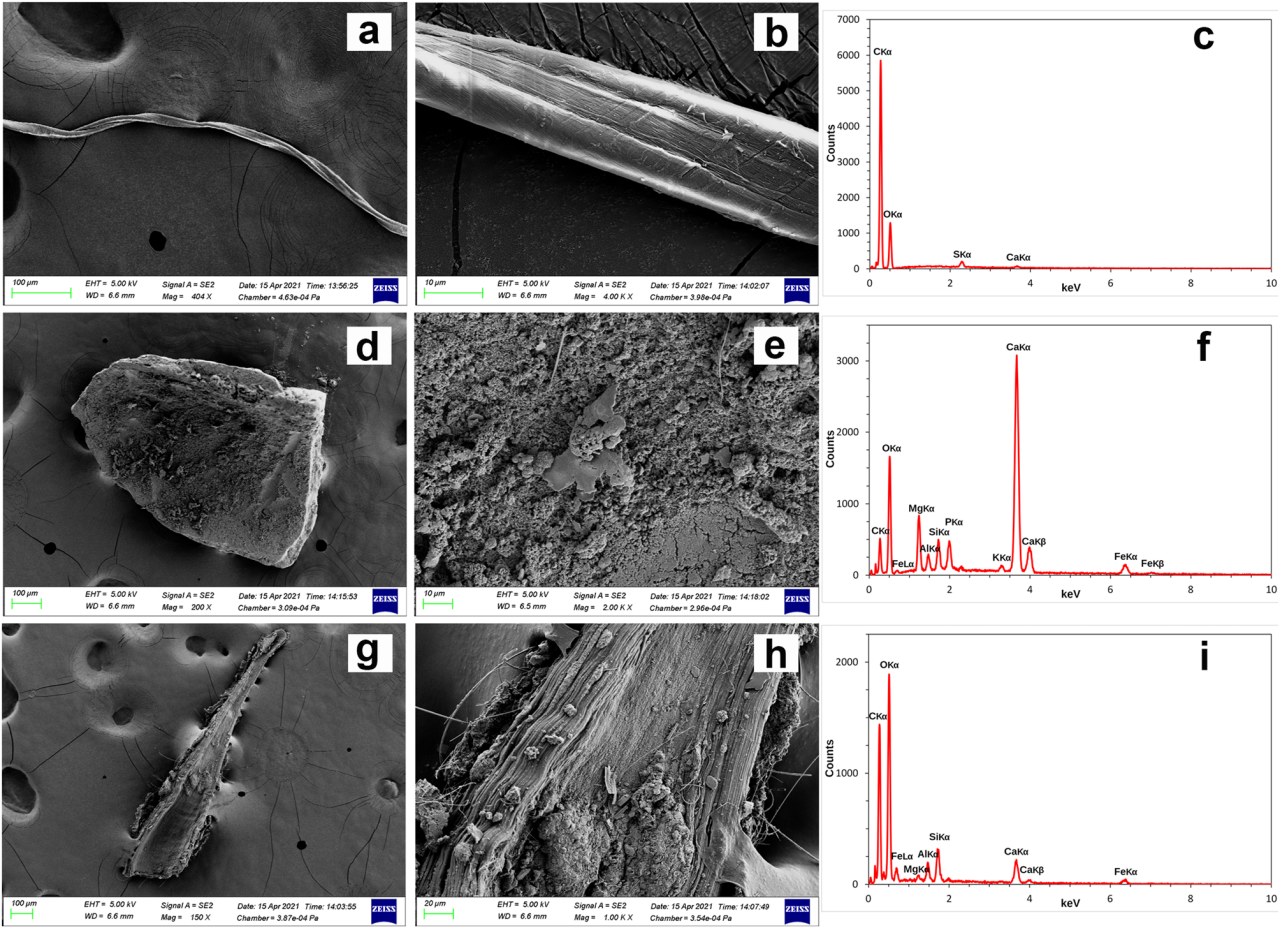
SEM/EDX images and microanalysis from non-plastic materials. SEM images (**a**,**b**,**d**,**e**,**g**,**h**) and spectra (**c**,**f**,**i**) of non-plastic materials obtained from the stomachal content of collected *Bursatella leachii* specimens. (**a**–**c**) natural cotton fibre. (**d**–**f**) Part of a mollusc shell made mainly of Calcium Carbonate. (**g**–**i**) Piece of plant organism with the cellulose as the main component.

Surface texture created by environmental exposure is one of the primary characteristics that can be used to screen for microplastics by electron microscopy. Morphological analysis of the microplastic particle surfaces often revealed degradation and abrasion signs, suggesting mechanical weathering processes^89,90^, which were observed in this study. SEM/EDX analyses provided high resolution pictures of the particles surface structure of the fibres and fragments analysed, as well as their elemental composition signatures. This information was used to screen for likely microplastics and rule out non-plastics. Since it is known from literature that the most common kind of plastics, as Polypropylene (PP) and Polyethylene (PE), show a strong Carbon EDX peak^90^, and considering that we are dealing with plastics extracted from an open environment, possibly affected by all the variety of existing plastics, we searched for spectra showing a significant concentration of Carbon to be the possible candidates for microplastics. Resultant spectra were compared with some reported by published studies carried out in laboratory and using previously known plastics polymers that were used as reference. Examples of spectra from canonical microplastic fragments and fibres are shown in Fig. 5 as well as spectra from different kind of plastic (Fig. 6) where it is easy to see the Carbon peak (C) and other few additional elements characteristic of other plastic types. Spectra obtained were characterized by a high variability that perfectly reflects the huge variety of plastic currently available from natural environment. SEM/EDX analysis from fragments and fibres which were determined to be non-plastic (such as natural fibres, mollusc’s shell fragments and debris of plant organisms) were also shown (Fig. 7).

## Discussion

Morphological and elemental composition analyses of the stomachal content of *Bursatella leachii* from a highly polluted environment revealed the presence of microplastic fibres and fragments in all the studied individuals. Despite increased international attention, investigating microplastic in environmental samples is a difficult task, because of its wide range of possible interactions with the living biota and because microplastic includes different organic polymers which can be chemically and mechanically modified by environmental factors like the weather, hydrodynamic forces, solar radiation, the presence of other contaminants, the occurrence of biofouling, etc.^91–96^. The complexity of the processes of cause/effect characterizing microplastic implies the use of different protocols each of them optimized for a specific target of study. Additionally, even if studying microplastic is nowadays of a central importance and several standardized protocols of extraction and analysis have been published^97–102^, the continuous search for the most effective or performing one is still ongoing^103–105^. Furthermore, even though considerable research effort focused on several target species, (mainly vertebrates from fish to mammals)^17,19,21^, few invertebrates were investigated so far, and, among them, filter-feeder molluscs (bivalves) were mainly studied^15,31,39,57^. To date, interactions between environmental microplastics and Heterobranchia remained neglected, perhaps due to the difficulties of studying small and infrequent animals that are characterized by soft and very delicate internal anatomy; however, bridging the knowledge gap on these benthic consumers—known to unceasingly explore soft and hard bottoms on the seafloor in search of food and with different trophic preferences—is indeed a highly promising challenge. In fact, the potential of marine Heterobranchia is high since these gastropods are characterized by unique defensive strategies like the ability to accumulate and, in most of the cases, modify, chemical active compounds obtained from the diet. Considering that microplastic is everlasting due to the absence of known multicellular organisms able to digest it, the capacity showed by marine Heterobranchia to modify foreign chemical molecules may be of great interest under a potential biotechnological perspective.

In this framework, we investigated stomachal content from *Bursatella leachii*, an Aplysiidae living in Mar Piccolo of Taranto (Ionian Sea, Mediterranean Sea), a coastal aquatic environment under high anthropogenic pressures, particularly exposed to plastic pollution (Fig. 1). The combination of the high-resolution SEM morphological observations with the EDX elemental composition of the debris was very useful to investigate and identify microplastics in the digestive trait of *B. leachii*. In fact, considering that microplastic can bind with other pollutants already present in the environment and taking into consideration that Mar Piccolo of Taranto host several anthropogenic pollutants, it can be hypothesised that the collected microplastic may show an atypical chemical composition, reflected by a non-canonical spectrum. This characteristic would eventually affect results from other kind of techniques like for example the FTIR analyses, since it is based on the perfect match between the spectra investigated and the canonical spectra of plastic already available in the reference public libraries. Being extracted from individuals living in a natural environment and not from ‘in laboratory study’, EDX spectra obtained were characterized by a high variability that perfectly reflects the vast diversity of plastic polymers now recorded in natural environments^48^.

The data presented here can be considered as preliminary, as they are based on dissection and analysis of six specimens only: a larger sampling will be required to corroborate and enlarge the value of these initial observations. However, the information gathered so far seems to be indicative of a consistent pattern. Firstly, fibres and small fragment particles were abundant in all the *Bursatella* stomachs analysed. This could be due to a selection made by the individuals with the exclusion of larger fragments and in favour of smaller ones or fibres stuck to the algae they feed on. Anyway, the dominance of fragments instead of fibre particles is of outmost interest and unexpected, since almost all previous studies on benthic animals report the opposite condition. This finding may corroborate the hypothesis of an active choice made by *B. leachii*, ingesting size- and shape-specific plastic debris and not just the most abundant ones, as usually happen for filter-feeders and/or non-selective detritivore organisms. In fact, fibres are expected to be copious in Mar Piccolo sediments, where they derive from abundant textile waste, degrading fishing lines, and particularly from the plastic nest nets widely used in mussel aquaculture (Fig. 1c–f), a major source of local contamination^24^. These preliminary data will represent baseline information for future comparative studies. Indeed, the analyses of high-res SEM images, together with the corresponding EDX spectra of the analysed fragments and fibres, highlighted the variability of the pool of debris that could be found in the field, with reference to already published information on compounds belonging to both natural and plastic materials. In fact, there are some natural materials that may resist the KOH digestion and therefore may require a future, in-depth analysis to distinguish them from the microplastic dataset and to avoid misidentifications.

Regarding the microplastic debris found in *B. leachii*, the canonical spectra that could be identified as plastic are reported in Fig. 5 both related to fragments and fibres. These spectra are consistent with the ones from literature as microplastic^90,106^. Among these, an important class is that of fibreglass which includes different kind of plastic all of them characterized by Carbon (C), Oxygen (O), and Silicon (Si)^107,108^ (https://www.nrc.gov/docs/ML0530/ML053040493.pdf) where the glass fibres are commonly added to reinforce plastic structures^109,110^. This specific kind of plastic is part of the components detected in the present study (Fig. 6). Apart from the elements discussed till now, other interesting and unusual elements were found like Titanium (Ti), Barium (Ba) and Zinc (Zn) (Fig. 6). These elements are used as additives in some types of plastics thus driving a more precise identification^111,112^. SEM/EDX microanalysis from fragments and fibres which were found to be non-plastic were also shown to be useful reference for further in situ studies focused on animals in natural environments (Fig. 7). In fact, among the samples that were non-plastic, there was a natural cellulosic fibre (cotton) characterized by a typical twisted morphology and the presence of peaks resembling those reported for cellulose (Fig. 7a–c)^113–115^.

Also, we detected debris containing a high concentration of calcium carbonate (CaCO_3_) (Fig. 7d–f), which is coherent with spectra reported for bivalves and gastropod shells^31,32,116,117^, tubes of sedentary polychaete worms^118^ and therefore not associated with plastic. The almost equal concentration of Carbon and Oxygen (C and O) elements detected in a single fragment (Fig. 7g–i), together with the characteristic external morphology, is related to the cellulose and, indeed, to plant organisms and not to plastic. Another important result for consideration must be the absence of Nitrogen (N) in the EDX spectra of our samples. This element in fact is one of the main components of biological compounds (containing proteins and/or peptide bonds) such as egg capsules of the gastropod *Rapana venosa* (Valenciennes, 1846)^119^ and eggshells of the Ascarididae nematode *Ophidascaris baylisi* Baylis (1921)^120^.

Investigating the variability among plastic materials is essential to fill the gap of knowledge still existing to date, avoiding misidentifications and errors in quantification of microplastic in the environments or into living organisms^91–96,121^. This last point is crucial and constitutes one of the main limits of the recent studies. In fact, most of them are carried out in laboratory and have used already known kinds of plastics which are therefore characterized by a known specific composition, while many other papers focused on plastic obtained by in situ studies but analysed using reference spectra from virgin plastic with known composition and eliminating those which are far from that reference. Anyway, the plastic debris found in a natural environment, and even more in a very polluted one, is not virgin plastic but instead a mixture of organic polymers plus a lot of different other elements that are added as additives or that are independently attracted by plastic due to chemical properties of the plastic itself. In fact, the microplastics found in the stomachs of *B. leachii* living in Mar Piccolo represent strong evidence of the high level of plastic contamination acting in this semi closed basin and the ease with which it enters the food chain as it is ingested by organisms that largely select the nutrients they eat. Interestingly, none of the stomachs analysed contained detritus, confirming that *B. leachii* is not a detritivore but selects plant organisms and a lot of other living organisms, in some way selecting them. We cannot exclude, therefore, that the microplastics were deposited on the surface of the prey ingested by the sea slugs. Anyway, it is interesting to note that once in the marine environment, microplastics are colonised by the ‘plastisphere’ as are collectively called the wide variety of microbial communities coating plastic debris and forming biofilms^121,122^. Considering that it was recently demonstrated the higher preference of some marine filter-feeders to eat microplastics coated with microbial biofilms instead of virgin microplastics, we can speculate the possible trophic preference of *B. leachii* for microplastics covered by cyanobacteria or other microbial biofilms. Anyway, this important ecological aspect is to date poorly known since most of the studies investigating the impacts of microplastics ingestion by aquatic organisms have currently used virgin plastic particles, which, however, do not reflect the real conditions of the sea^121^ as the results here reported have also demonstrated. Another consideration can be made regarding the fact that plastic was found in all the specimens analysed independently if they had a lot of food in the digestive apparatus or not. This could be evidence of the possible persistence of plastic that would not be excreted with faeces. Taking into consideration that Heterobranchia have the capability to accumulate exogenous material and chemically modify it for defensive purposes, this observation becomes even more interesting. Further in-depth studies are needed to fulfil the gap of knowledge on this intriguing biological capability with potential highly innovative applications.

In conclusion, this work constitutes a baseline study useful for future in-depth investigations on the diet and faecal contents and for future comparisons with plastics contents from digestive traits of other taxa and with EDX spectra from other materials of difficult identification. Furthermore, it could be a reference database on the composition of locally originated plastics in the Mar Piccolo (due to its typical hydrology) that will be useful to simulate possible future scenarios on other polluted coastal areas and to predict the potential outcome of negative impacts on the biota.

## Supplementary Information

Supplementary Information.

## Data Availability

All data generated or analysed during this study are included in this published article (and its Supplementary Information files).
